# Loneliness and Risk of Post-Stroke All-Cause Dementia: A Cohort Study

**DOI:** 10.1192/j.eurpsy.2026.10724

**Published:** 2026-07-31

**Authors:** Y. Chen, M. Li, Y. Ou, Y. Y. Liang

**Affiliations:** 1 Department of Psychology, The University of British Columbia, Vancouver, Canada; 2 Institute of Psycho-neuroscience, The Affiliated Brain Hospital, Guangzhou Medical University; 3 Institute of Psycho-neuroscience, The Affiliated Brain Hospital, Guangzhou Medical University, Guangzhou, GuangZhou, China

## Abstract

**Introduction:**

Up to 10–30% of stroke survivors develop dementia. Loneliness has been shown to be a psychosocial risk factor for developing stroke and cognitive impairment in the general population. Stroke survivors are more likely to experience loneliness. However, the contribution of loneliness to post-stroke dementia risk remains unclear.

**Objectives:**

We aimed to investigate the association between loneliness and incident dementia in stroke survivors.

**Methods:**

We included 7,267 UK Biobank (No. 93604) participants with prior stroke (mean age =60.5 ±6.9 years, 55.6% male) without baseline dementia. Loneliness was assessed via two self-report questions (frequency of feeling lonely and frequency of confiding in someone close) and defined as a score of 2 (lonely) vs <2 (not lonely). Incident post-stroke dementia (all-cause) was ascertained from hospital records and death registries. We used Cox cause-specific hazards models to estimate hazard ratios (HRs) adjusted for demographics, socioeconomic status, comorbidities, health behaviours and physiological covariates. We also performed a relative importance analysis to rank risk factors. We calculated the Percentage of Excess Risk Mediated (PERM) by grouping explanatory covariates into categories, such as medication, behaviors, and comorbidities.

**Results:**

Over a median 10.2-year follow-up, 290 stroke survivors developed dementia. Loneliness was significantly associated with a higher risk of dementia (fully-adjusted HR 1.67, 95% CI: 1.12–2.49). In a risk-factor ranking, loneliness was the second most important predictor of post-stroke dementia, ranking ahead of conventional factors such as hypertension and obesity. The association between loneliness and post-stroke dementia was magnified in stroke survivors (HR: 1.67, 95% CI: 1.12–2.49) compared to individuals without stroke (HR: 1.22, 95% CI: 1.07–1.39). 25% of the excess dementia risk associated with loneliness was attributable to diabetes-related factors, with the remainder largely explained by medication, behaviors, comorbidities, and other covariates.

**Conclusions:**

Loneliness is an independent risk factor for developing dementia among stroke survivors. This association is partly mediated by modifiable behavioral and physiological pathways. Our findings highlight loneliness as a prominent, yet addressable, risk factor for post-stroke dementia, underscoring the importance of screening for feelings of loneliness in stroke aftercare.

**Disclosure of Interest:**

None Declared

